# High yield extraction of pure spinal motor neurons, astrocytes and microglia from single embryo and adult mouse spinal cord

**DOI:** 10.1038/srep16763

**Published:** 2015-11-18

**Authors:** Marie-Josée Beaudet, Qiurui Yang, Sébastien Cadau, Mathieu Blais, Sabrina Bellenfant, François Gros-Louis, François Berthod

**Affiliations:** 1 Centre LOEX de l′Université Laval, Centre de recherche du CHU de Québec, Québec (Québec), Canada; 2 Département de Chirurgie, Faculté de Médecine, Université Laval, Québec (Québec), Canada

## Abstract

Extraction of mouse spinal motor neurons from transgenic mouse embryos recapitulating some aspects of neurodegenerative diseases like amyotrophic lateral sclerosis has met with limited success. Furthermore, extraction and long-term culture of adult mouse spinal motor neurons and glia remain also challenging. We present here a protocol designed to extract and purify high yields of motor neurons and glia from individual spinal cords collected on embryos and adult (5-month-old) normal or transgenic mice. This method is based on mild digestion of tissue followed by gradient density separation allowing to obtain two millions motor neurons over 92% pure from one E14.5 single embryo and more than 30,000 from an adult mouse. These cells can be cultured more than 14 days *in vitro* at a density of 100,000 cells/cm^2^ to maintain optimal viability. Functional astrocytes and microglia and small gamma motor neurons can be purified at the same time. This protocol will be a powerful and reliable method to obtain motor neurons and glia to better understand mechanisms underlying spinal cord diseases.

*In vitro* culture of purified motor neurons (MNs) is a useful approach to investigate numerous aspects of neuronal behavior, such as developmental biology, cell physiology, genomic, susceptibility to neurotoxins, etc. Furthermore, MN cultures are particularly important in the study of neurodegenerative diseases such as amyotrophic lateral sclerosis (ALS), which is characterized by MNs degeneration^1^. However, the mechanism of MN degeneration still remains unresolved despite the discovery that mutations in the superoxide-dismutase (SOD1) caused the disease in 1–2% of ALS patients, and the development of transgenic mice overexpressing the mutated protein (SOD1G93A and SOD1G37R mice) and recapitulating the disease symptoms^2^,^3^. In these mouse models, massive death of motor neurons in the ventral horn of the spinal cord and loss of myelinated axons in ventral motor roots can be observed, ultimately leading to paralysis, muscle atrophy and death.

The easiest way to obtain MNs from mice is to collect them from embryos between the 12^th^ and 14^th^ day of development (E12 to E14), when MNs are mature enough to be collected.

MNs can be isolated from the other spinal cord cells due to their larger size by centrifugation through a gradient density^4^,^5^,^6^. To obtain large amount of pure embryonic MNs from a single wild type mouse (typically 20,000 to 100,000 cells per embryo), it has been previously shown that MNs must be pooled together from typically 5 to 8 embryos^5^,^7^. However, considering the reduced fertility of homozygous ALS transgenic SOD1 mice, the isolation of large amount of pure embryonic motor neurons using transgenic pregnant homozygous SOD1 mice is practically impossible. Indeed, SOD1G93A female homozygous for the mutated SOD1 are poor breeders and rarely produce more than one litter before the onset of the disease. It is therefore recommended to breed hemizygous male carrier to non-carrier wild-type females at each generation. In this case, the genotype of each fetus becomes uncertain, excluding out the possibility to pool together cells obtained from different embryos. In order to allow the culture of an adequate number of MNs expressing the desired genotype from mouse embryos, it would therefore be necessary to greatly increase MNs enrichment yields and to ultimately process each spinal cord collected from single embryo individually. Attempts have been previously described for time-consuming MNs extraction protocols from single embryos using immune-affinity purification. These protocols typically use a p75 (NTR)-antibody-based cell-sorting panning technique, p75 being an extracellular protein exclusively expressed in the spinal cord by MNs^8^,^9^,^10^,^11^. Although this method yielded up to 80% pure spinal MNs, it was not possible to isolate more than 15 000 cells from individual embryo^10^. However, since the use of anti-p75 antibodies is highly expensive, an alternative approach using a lectin with high affinity to p75 has been establish, with the same cell-sorting panning strategy leading to lower yields than with p75 antibodies^11^. These panning approaches may necessarily yield lower number of MNs and be more expensive than isolation based on the MN large size, which in turn could lead to lower purity. If MN purity can be kept high with the gradient density approach, then its simplicity, reduced cost and higher yield are very advantageous.

Beside extraction of embryonic MNs, the isolation of these cell populations using adult mouse spinal cords has been also shown to be highly challenging^5^,^12^,^13^. Therefore, alternative approaches were used, such as the microinjection of the mutant SOD1 protein in normal mouse MNs^14^, or differentiation of MNs from stem cells originating from human or transgenic mice^15^,^16^,^17^. All these approaches have drawbacks, such as a difficulty to reproduce a clear ALS phenotype *in vitro* or the introduction of intermediary steps that may interfere with the disease mechanism (microinjection could induce stress and damage neurons). Thus, improvements in the extraction, isolation and purification protocols of adult spinal MNs would be highly beneficial for the study of MN development, maintenance and degeneration.

Furthermore, existing MNs extraction methods, either using mouse embryos or adult mouse spinal cords, do not allow for parallel isolation and purification of primary motor neurons, microglia and astrocytes from the same tissue sample. The parallel isolation of all these different cell populations will be of particular importance to understand SOD1-linked pathogenic mechanism considering the well known non-cell autonomous paradigm described in ALS^18^. Moreover, spinal astrocytes represent a heterogeneous cell population that participates in a variety of physiological processes in the central nervous system^19^,^20^,^21^. Consequently, the possibility to extract, purify and culture adult astrocytes and microglia from mice at different specific disease stages would be essential to better understand neurodegenerative mechanisms.

Astrocytes can be commonly obtained from glia cultures devoid of microglia using embryos, new born mouse spinal cords or brain of young rodents^22^,^5^,^23^. However, astrocytes obtained from new born pups do not possess all the functional properties of adult astrocytes since they do not have processes and have a flat square-like shape instead of ramified stellar shape, and their gene expression profile and proliferating rate differs significantly from adult *in vivo* astrocytes^23^. Mature astrocytes were previously considered to be impossible to obtain from old rodents^23^. In addition, GFAP expression is not a specific marker for the assessment of functional mature astrocytes^21^,^24^. Spinal astrocytes represent a heterogeneous cell population^19^,^20^,^21^ that participates in versatile physiological processes in the central nervous system. Consequently, astrocytes have to be screened for a large variety of markers to be adequately characterized *in vitro*. The possibility to extract, purify and culture adult astrocytes from old mice at different specific disease stages such as in ALS would be extremely valuable to better understand neurodegenerative mechanisms.

There are two types of microglia in healthy central nervous system (CNS), amoeboid (activated) and mature ramified (surveying non-activated). These two types of microglia are distinct phenotypically and functionally. Amoeboid microglia are usually isolated by agitating off from glia cultures^13^,^19^,^21^,^24^. Both amoeboid and ramified microglia can be directly purified through density gradient centrifugation^25^,^26^ or magnetic isolation^27^,^28^ of CNS tissue homogenous suspension. But it is very difficult for primary adult microglia to survive and expand alone without astrocyte in culture even supplemented by growth factors^29^,^30^ and optimized culture medium^26^. In addition, most protocols for adult microglia extraction have been designed for the brain, instead of the spinal cord^26^,^31^.

The aim of this project was therefore to design a protocol enabling high and reproducible MN extraction yields from one single mouse embryo and to adapt it to adult mouse spinal cords while keeping a high degree of cell purity. In addition to the extraction, purification and culture of alpha MNs, this protocol will allow obtaining adult spinal cord stellar or protoplasmic astrocytes, microglia and gamma small MNs from the same tissue sample.

## Results

### Parallel isolation of pure spinal MNs and glial cells extracted from mouse embryos

**Figure 1:**
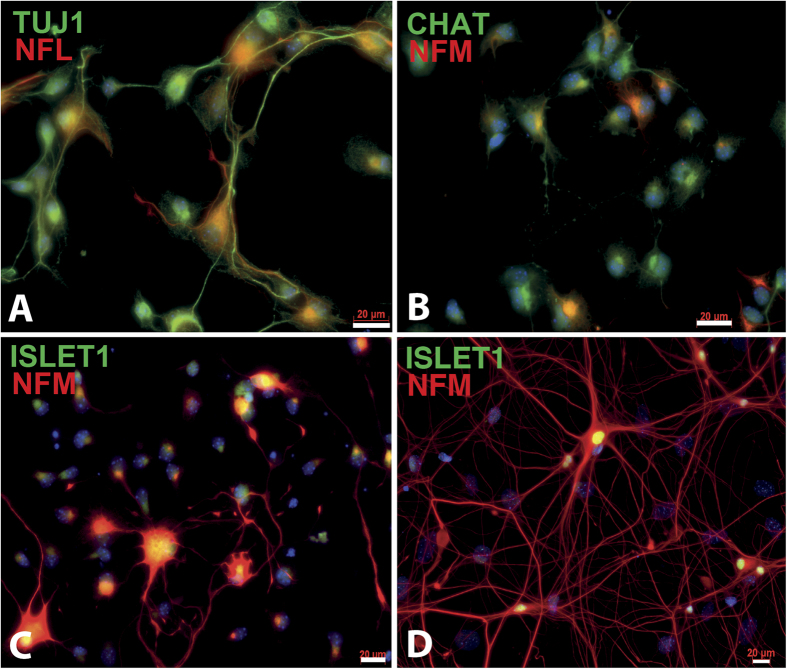
Characterization of purified MNs obtained from E14.5 embryo spinal cord of CD-1 mice. MNs were extracted from E14.5 embryos and characterized by immunofluorescence staining using the TUJ1 (**A**) in green), NFL ((**A**), in red) and NFM ((**B–D**) in red) neuronal markers and the CHAT (**B**) in green) and Islet1 ((**C,D**) in green) specific MN markers after 2 days of culture on a poly-D-lysine coated cover glass (200,000 cells/cm^2^). NFM (in red) and Islet1 (in green) positive cells with long neurites can be observed after cultivating MNs for 14 days (**D**). Scale bars 20 μm.

In order to extract at the same time MNs, astrocytes and microglia from mouse embryos, we have used in the past a discontinuous density gradients system^5^. This method allowed us to extract in average 100,000 of primary MNs per E12.5 CD-1 mouse embryo, along with astrocytes and microglia with high purity (>98%). An optimized and simplified approach using this purification method allowed us to obtain higher recovery rates of purified embryonic MNs extracted from one single embryo. We recovered 1.4 million MNs and 473,000 glial cells per CD-1 mouse embryo (56 embryonic spinal cords were isolated from 6 pregnant females) at E13.5 (Table 1). This number was increased to 2 million MNs and 891,000 glial cells by collecting E14.5 mouse embryonic spinal cords (48 embryonic mouse spinal cords were isolated) while 2.7 million MNs and 855,000 glial cells in average were collected from Hxlb9-GFP transgenic E14.5 embryos (57 embryonic mouse spinal cords were isolated) (Table 1). Better recovery yields from E14.5 embryos were therefore obtained compared to E13.5 probably because embryonic MNs collected at E14.5 were larger and can be more easily separated using density gradient. In order to confirm the cellular identity, immunofluorescence and morphological analysis were done using specific molecular markers for each extracted cellular types. Cells collected at the medium/gradient interface all expressed the neuronal marker TUJ1 as well as the motor neuron markers NFM, ISLET1 and CHAT confirming the motor neuron identity (Fig. 1). Moreover, abundant NFM/ISLET1-positive MN neurite outgrowth can also be appreciated when the collected MNs were cultured for 14 days (Fig. 1D). The mean value of MN purity, data calculated by immunofluorescence from 49 E14.5 CD-1 embryonic spinal cords extracts and from 19 E14.5 Hxlb9-GFP transgenic embryos, was 95% and 97%, respectively (Table 1). These results were further confirmed by flow cytometry analysis after staining of CD-1 embryo motor neurons with ISLET1 showing 93% of Islet1-positive cells (Table 1). Astrocytes and microglia can also be obtained from E14 mouse embryos using this protocol, as shown by immunostaning with the GFAP astrocyte marker and IBA1 microglial marker (Supplementary Fig. S3).

### Isolation of pure MNs extracted from mouse adult spinal cords

**Figure 2:**
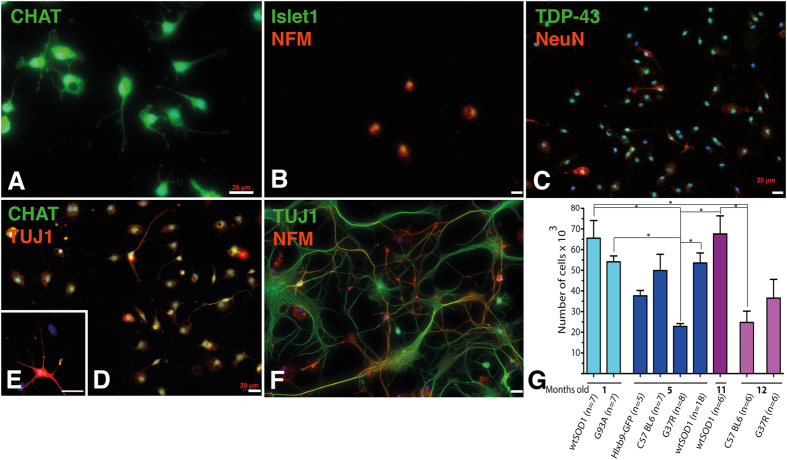
Characterisation of purified MNs obtained from adult spinal cord. MNs (with a 90% purity) were extracted from 5-month-old SOD1^G93A^ mice at disease onset, and cultured for one day (**A–D**) and 15 days (**E,F**), and characterized for expression of CHAT (in green, (**A,D,E**)), Islet1 (in green, (**B**)) and NFM (in red, (**B,F)**), TDP-43 (in green, (**C**)) and NeuN (in red, (**C**)), TUJ1 (in red, (**E,D**), and in green, (**F**)) and NFM (in red, (**F**)) (200,000 cells/cm^2^). The yield of MNs obtained from different mouse lineages 1, 5, 11 and 12 months after birth was quantified and showed significant variations from 19 000 to 65 000 MNs (*p < 0.05) (**G**). Scale bars 20 μm.

Our optimized MNs isolation procedure also allowed for the extraction of pure MNs extracted from individual adult spinal cord (Fig. 2). Purified adult MNs obtained from SOD1G93A transgenic mice stained positive for the neuronal markers ISLET1, TDP-43, TUJ1, NeuN, NFM and CHAT at day one post-extraction (Fig. 2A–D) and at day 15 (Fig. 2E,F). A purity over 90% +/− 5% (mean +/− STDEVA; n = 10 extractions), calculated by counting the number TUJ1/CHAT positive cells over to total amount of nuclei, was obtained with this protocol. Various transgenic mice were tested, C57 BL6 mice corresponding to the original mouse background used to generate the other mouse types, wtSOD1 mice overexpressing the normal human SOD1, SOD1^G93A^ mice overexpressing the human mutated SOD1 to induce ALS, SOD1^G37R^ mice overexpressing the human mutated SOD1 to induce ALS (with a lower SOD1 expression than G93A) and Hlxb9-GFP mice expressing GFP under the Hb9 promoter (to generate GFP-expressing MNs). The number of MNs extracted from adult mice from 1 to 12 months after birth varied from 20 000 to 65 000 MNs per mouse depending on the type of mouse lineage and the mouse age (p < 0.05) (Fig. 2G and Table 2).

### MNs viability in culture

Our optimized MN extraction protocol allowed *in vitro* MN cultivation for at least 21 days post-extraction from individual mouse embryo (Fig. 1C). Since we noticed that MN viability highly depends on the plating density, we have assessed different plating conditions to identify the optimal density to preserve MNs viability. Seeding MNs at a concentration lower than 100,000 cells/cm^2^ resulted in decreased cellular viability shortly after 3 days of culture (Supplementary Fig. S1). Furthermore, seeding MNs at a concentration of 50,000 cells/cm^2^ or lower induced drastic cellular death (over 75%) and limited neurite outgrowth (Supplementary Fig. S1). Ninety percent cell death was even observed after only 3 days of culture when MNs were seeded at a concentration of 10,000 cells/cm^2^ (Supplementary Fig. S1). In contrast, only 25% of cellular death was observed after 6 days of culture when MNs were seeded at a concentration of 100,000 or 200,000 cells/cm^2^ (Supplementary Fig. S1).

### Transfection of MNs

Gene transfer techniques using primary MNs to study basic mechanisms of development, axon growth and pathfinding, or pathogenic mechanisms underlying motor neuron diseases have been shown challenging. Complex microinjections or magnetotransfert techniques are usually used. However, as shown in Supplementary Fig. S2, our MNs extraction protocol allows the introduction of genetic materials into *in vitro* MN cell culture using a simple lipid-based transfection reagent such as lipofectamine with few modifications from manufacturer’s instructions. We were indeed able to successfully transduce the primary cultured MNs extracted from Hxlb9-GFP mice with a plasmid to induce expression of ds-Red for 21 days of culture (Supplementary Fig. S2).

### Isolation of adult astrocytes and microglia extracted from 5-month-old mice spinal cord

**Figure 3:**
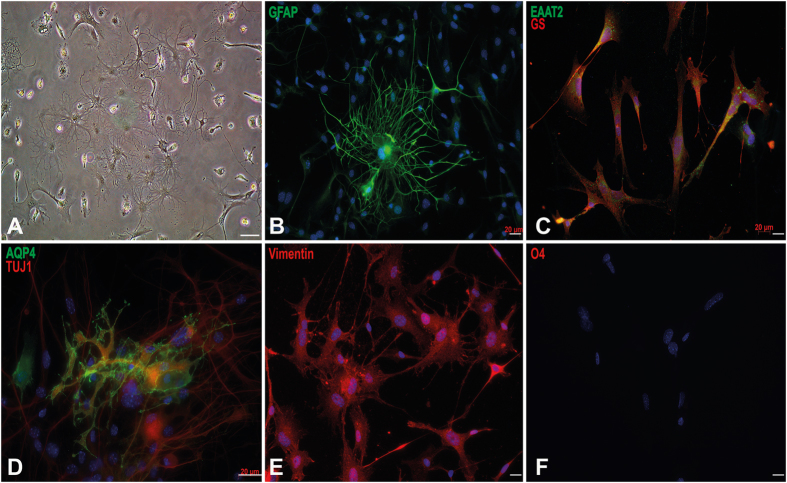
Characterisation of purified astrocytes obtained from adult spinal cord of 5-month-old SOD1^G93A^ mice. Phase contrast microscopy of extracted cells after 7 days of culture showing astrocytes, microglia and small MNs (**A**,**D**). Identification of purified astrocytes after 14 days of *in vitro* maturation by immunofluorescence staining using GFAP (In green; (**B**)), EAAT2 (in green) and GS (in red) (**C**), aquaporin-4 (AQP4) in green and TUJ1 in red (**D**), Vimentin in red (**E**), and O4 in red (**F**). Cells were negative for O4 marker excluding them as oligodendrocytes. Scale bar 20 μm.

As mentioned above, adult astrocytes and microglia are difficult to extract from adult spinal cord. In spite of these repeatedly reported difficulties, our extraction protocol allowed us to isolate different cellular types including astrocytes as well as microglia from adult mouse spinal cords. Actually, stellar astrocytes, protoplasmic astrocytes, amoeboid microglia and neurons can be easily isolated from 5-month-old WTSOD1 as well as SOD1G93A mouse spinal cords (Fig. 3A). Immunocytochemistry also revealed the presence of large ramified astrocytes expressing specific GFAP, Aquaporin 4, Vimentin, EAAT2 and GS (glutamine synthetase) molecular markers (Fig. 3B–F). Note that these cells do not express the oligodendrocyte O4 marker, confirming their astrocytic cellular identity.

**Figure 4:**
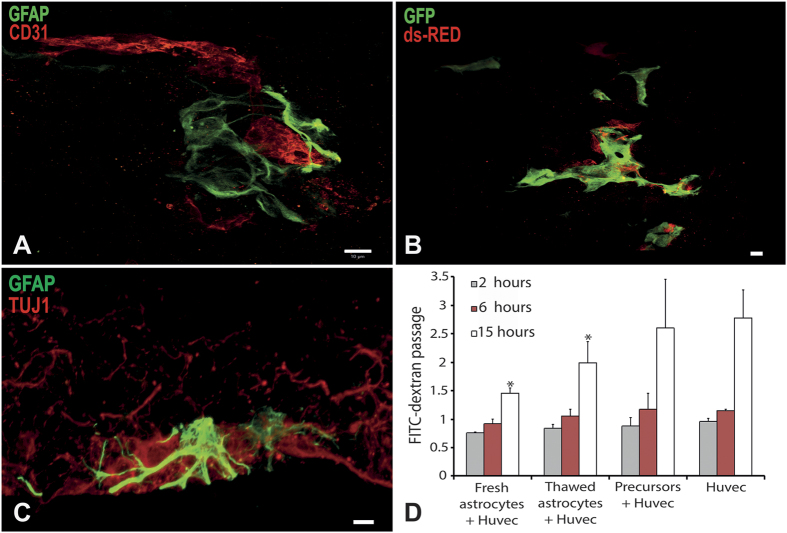
Characterisation of astrocyte functionality obtained from adult spinal cord of 5-month-old wtSOD1mice. Astrocytes were cocultured with endothelial cells and fibroblasts in a three-dimensional culture system in which capillary-like tubes form (**A,B**). Astrocytes were characterized by GFAP expression in green (**A,C**), or transduced to express ds-Red (**B**). Endothelial cells were detected by CD31 expression in red (**A**) or transduced to express GFP in green (**B**). Astrocytes were cocultured with MNs in a three-dimensional culture system promoting axonal migration. Astrocytes were identified using GFAP expression in green, and MNs using TUJ1 expression in red (**C**). The capacity of astrocytes to decrease endothelial cell permeability was assessed by quantification of FITC conjugated dextran diffusion through an endothelial cell monolayer, compared to spinal cord-derived oligodendrocyte progenitor cells as a control. Astrocytes significantly decreased FITC-dextran diffusion through endothelial cell monolayer after 15 hours of contact compared to endothelial cells alone or endothelial cells cocultured with oligodendrocyte progenitor cells (p < 0.005; n = 3). Scale bar 10 μm.

In order to test the functionality of the isolated astrocytes, i.e. their capacity of interacting with microvessels to form functional astrovascular unit, we have used a validated co-culture system allowing three dimensional culture of astrocytes and endothelial cells, in which capillary-like tubes can be formed^32^,^33^. Interestingly, immunocytochemistry revealed that astrocytes, isolated via our technique, closely interacted with endothelial cells, forming astrocytic foot processes affecting vascular permeability (Fig. 4). Indeed, the vascular permeability, tested using FITC-conjugated dextran diffusion through a confluent endothelial cell monolayer after 15h of incubation with the isolated astrocytes, was found to be significantly decreased therefore indicating that our extraction protocol allowed to reproduce *in vitro* one important function of the blood-brain-barrier.

**Figure 5:**
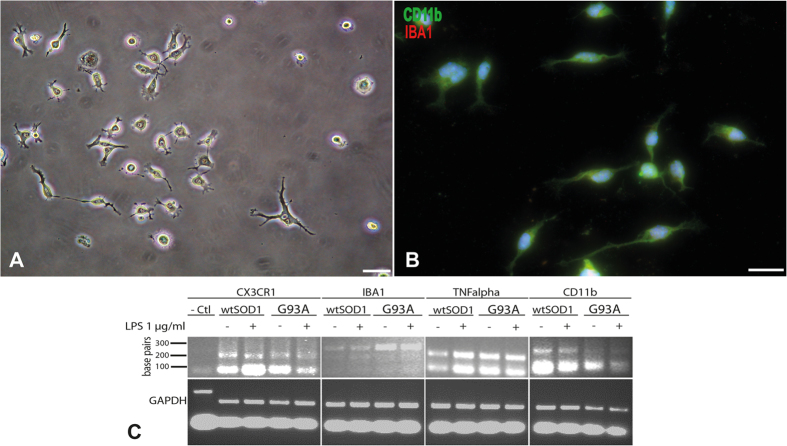
Characterisation of microglia purified from adult spinal cord of 5-month-old mice. Phase contrast microscopy of purified microglia cells after 14 days of culture (20,000 cells/cm^2^) (**A**). Immunocytochemical characterization of purified microglia using CD11b in green and IBA1 in red (**B**). Scale bar 20 μm. Qualitative PCR showing expression by microglia from wtSOD1 and SOD1^G93A^ mice of specific markers such as CX3CR1, IBA1, TNFalpha and CD11b, after activation or not with LPS (**C**). Urothelial cells were used as negative control.

Adult microglia can also be obtained and identified by immunocytochemistry as being IBA1 and CD11b-positive with this extraction protocol (Fig. 5B). The identity of these cell population was also further confirmed and functionally characterized by quantitative RT-PCR in the presence or absence of lipolysaccharide (LPS) using microglia specific PCR primers sets (IBA1, CD11b, CX3CR1 and TNFα) (Fig. 5C).

### Extraction and culture of small gamma MNs from 5-month-old mice

**Figure 6:**
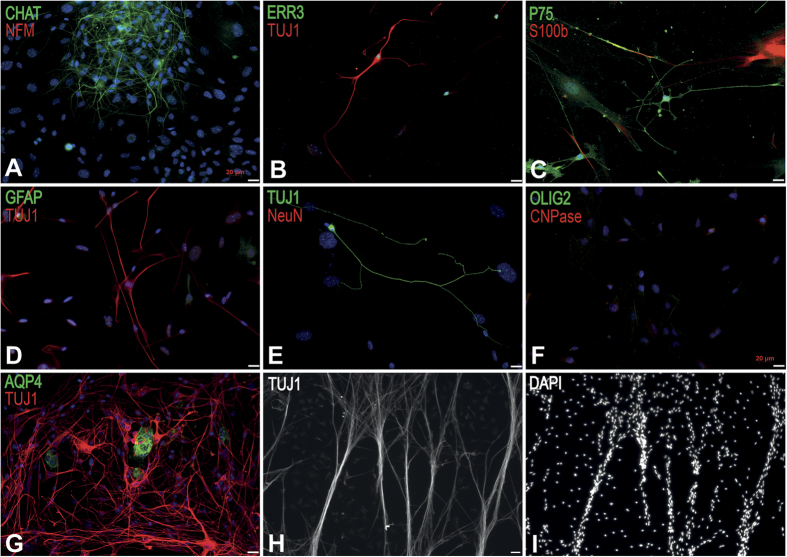
Characterisation of small gamma MNs from adult spinal cord from 5-month-old wtSOD1mice. (**A**) Identification of extracted small gamma MNs by immunofluorescence staining after 21 days of culture using CHAT in green and NFM in red (**A**), ERR3 in green and TUJ1 in red (**B**), P75 in green and S100b in red (**C)** (50,000 cells/cm^2^). The TUJ1-positive MNs did not express GFAP (in green, (**D**)), NeuN (in red, (**E**)), AQP4 (in green, (**G**)), OLIG2 (in green) and CNPase in red (**F**). Nuclei were stained in blue with DAPI (**A,D,E,I**). The TUJ1-positive MNs formed aligned nerve bundles after 3 months in culture (**H**,**I**). Scale bar 20 μm.

Small gamma MNs can also be obtained from the glia fraction using our extraction protocol (Fig. 6). Gamma MNs were characterized by immunocytochemistry using molecular markers CHAT, ERR3, TUJ1, P75, NeuN, NFM, GFAP, S100b, OLIG2, CNPase and AQP4. Indeed, gamma MNs can be discriminated from alpha MNs and interneurons because of their smaller size, their ERR3 specific expression but their absence of expression of NeuN^34^. These MNs, either extracted from wild type or SOD1^G93A^ mice were very stable in long-term culture and organized into fiber bundles after 3 months of culture maturation (Fig. 6H,I).

## Discussion

We described here an optimized extraction and purification protocol allowing recovering in parallel high yield of pure alpha and gamma MNs, astrocytes and microglia from both mouse embryo and adult spinal cords^5^. This method is based on a previous protocol we developed to obtain mouse embryonic MNs using mild trypsin dissociation of spinal cord cells and purification of MNs with discontinuous step density gradient centrifugations. The purpose of this previous protocol was to obtain highly purified MNs, but not to optimize cell yields^5^. Indeed, the enriched MNs band was only collected at the 1.04 g/mol interface to maintain high purity. However, the 1.06 g/mol interface contains up to 50% percent of MNs, and MNs are also present between each phase of density gradient contributing to low extraction yields. In addition, it has been also reported that trypsin is not the optimal enzymatic method for cell extraction from nervous system tissues^35^,^36^. Consequently, to increase MNs yield and viability, we choose to dissociate cells using papain and to collect MNs in a one step gradient phase without BSA cushions and without DNase digestion.

More than 2 millions MNs can be obtained using this improved protocol from the spinal cord of a single mouse embryonic CD1 as well as transgenic Hxlb9-GFP (which express GFP upon activation of the reporter gene Hb9, specific for motor neurons) while keeping purity higher than 95%. In comparison, the recovery yield of other published protocol were dramatically inferior with less than 15,000^10^ and 100,000^5^ recovered MNs. Our higher recovery yield becomes critical since we showed that plating density is crucial for MN survival in culture, with an optimal density of 200,000 cells/cm^2^ to increase MN survival.

To summarize these improvements:

- We used E14.5 embryos instead of E12.5 allowing us to obtain 2 times more MNs.
- We use papain instead of trypsyn allowing for a better MN survival.
- Cell separation was performed using a slower centrifugation allowing for a much better separation between glial cells and MNs while using a one layer gradient.
- MNs were cultured at a density of 200 000 cells/cm2 for better long-term survival.

This new MN extraction protocol from a single embryonic mouse spinal cord is an asset to study MN development and disease. It will allows to perform individual extractions of MN from each embryo obtained from heterozygous transgenic mice, with enough cells to perform a whole experiment after embryos have been genotyped.

Moreover, this improved protocol, when applied to adult mouse spinal cord, allowed to extract and purify more than 30,000 MNs from 5-month-old mice, instead of only 500 in previous protocols^13^. From the same spinal cord sample, it was also possible to obtain small gamma MNs, astrocytes and microglia. All cells can be extracted and purified independently and maintained in long-term culture. Microglia and astrocytes, but not MNs can be frozen for banking. There was no apparent distinction between wtSOD1 or SOD1^G93A^ glial cells and small gamma MNs except the highest number of microglia purified from SOD1^G93A^ mice.

Since the majority of neurodegenerative diseases appeared with aging in humans, being able to obtain MNs and glial cells from adult mice at different stages after disease onset will be a valuable strategy to better understand the pathological mechanisms of these diseases.

Although it has been reported that adult microglia can be amplified and cultured in long term, those microglia were larger that 50 μm, instead of usually 10 μm, and had a different morphology than expected suggesting changes in their properties^31^. Our protocol allowed us to obtain 5-month-old microglia with an adequate 10 μm size and expressing the appropriate markers. Their weak response to LPS may be due to *in vitro* culture for 2 months before treatment. Chock and coworkers have shown that primary microglia from new born pups maintained two weeks in culture poorly respond to LPS compared to those maintain for only 3 days^37^.

In conclusion, this improved protocol of spinal cord cell extraction allowed for the first time to obtain more than 2 millions MNs from a single embryo, and more than 30,000 MNs from a single 5-month-old mouse. Moreover, it allowed obtaining and maintaining in long-term culture small gamma MNs, astrocytes showing *in vitro* functional capacities and microglia from the same spinal cord sample

## Materials and Methods

(see the step by step protocol in supplementary materials)

### Isolation of spinal cords

All experiments were performed in accordance with relevant guidelines and regulations of the Canadian Council on Animal Care, and were approved by the Laval University Animal Care Committee. Embryo spinal cords were obtained from pregnant CD-1 and Hlxb9-GFP mice (Jackson Laboratories) and adult spinal cords came from B6SJL-Tg (SOD1^G93A^) 1Gur/J, from B6SJL-Tg (wtSOD1) 2Gur/J or from wild type B6SJL, B6SJL-Tg (SOD1^G93A^) 1Gur/J, B6SJL-Tg (wtSOD1) 2Gur/J, C57BL6, C57BL6-Tg (SOD1^G37R^) and C57BL6-Tg (Hlxb9-GFP) background mice (Jackson Laboratories). Embryo spinal cords were extracted in sterile conditions under a dissecting microscope (Nikon, Mississauga, Canada), using small forceps into cold Leibovitz’s L-15 medium (Life Technologies) with 25 μg ml^−1^ penicillin-gentamycin (Sigma Chemicals). Dorsal root ganglia (DRG) were cut off from embryonic spinal cord using scalpel blade. Isolated embryonic spinal cords were transferred individually into a 12 wells plate, identified and kept in cold L-15 medium.

Adult spinal cords were isolated by cutting the vertebrate column with a strait scissor in front of the back legs and before medulla oblongata and extruded using a syringe filled with cold medium with 18G needle (BD Biosciences) into cold complete DH medium containing 36.54 mM NaHCO3 (Fisher Scientific), 0.18 mM L-adenine (Fisher Scientific), 312.5 μl L^−1^ 2N HCL (Fisher Scientific), 10% of fetal calf serum (Hyclone) and 25 μg ml^−1^ of penicillin-gentamycin (Sigma Chemicals). The adult spinal cords were transferred into cold complete DH medium.

### Cell extraction and separation

Individual spinal cords were transferred into 2 mg ml^−1^ papain (Worthington) dissolved into HBSS 1× (Life Technologies), cut into small pieces and digested for 20 min at 37° for embryonic spinal cords and for 30 min for the adult ones. Cells were extracted and isolated from pieces of tissue by adding cold medium to the digested tissues with 6 repetitions of gentle trituration using middle speed pipetman and transferred into tubes. This step was repeated trice and cells were centrifuged at 280 g for 10 min at 4 °C for MN extraction or at 300 g for glia. Adult glial cells were ready to plate after this step. MN pellets were resuspended in 6 ml of cold L-15 and laid over a 1.06 g ml^−1^ Nycoprep density solution (Axis-Shield)^4^ and spun at 900 g for 20 min at 4 °C without brake in a swinging bucket centrifuge. MN were collected at the interface of the Nycoprep solution and poured in a new 50 ml collection tube. If needed, glial cells were retrieved from the pellet. Each MN collecting tube was filled with cold L-15 and each glia ones with 10 ml of complete DH medium. MN cells were counted at this step. MN collecting tubes were centrifuged at 425 g for 10 min in a swinging bucket centrifuge at 4 °C and glia collecting tubes were centrifuged at 300 g for 10 min.

### Cell culture

MN pellets were gently resuspended at 200,000 cells/cm^2^ in Motor neuron medium containing one-to-one ratio of complete DH medium mixed with neurobasal A medium (Life Technologies) supplemented with 0.5 × B-27 (Life Technologies), 0.24 mM L-glutamine (Life Technologies), 0.2 μg ml^−1^ hydrocortisone (Calbiochem), 2.5 μg ml^−1^ insulin (Sigma Chemicals), 10 ng ml^−1^ human recombinant NT3 (Feldan), 10 ng ml^−1^ human recombinant GDNF (Feldan), 10 ng ml^−1^ human recombinant BDNF (Feldan) and 25 ng ml^−1^ human recombinant CNTF (Feldan)^5^. MNs were allowed to attach in a humidified 37 °C incubator for 1 h, washed twice with warm neurobasal medium (Life Technologies) and cultured in 500 μl of Motor neuron medium per 100,000 cells on plastic flasks coated with 10 μg ml^−1^ poly-D-lysine. Adult glia and gamma MNs were resuspended in Fibroblast-conditioned DH medium. For this medium, 6 millions of human new born foreskin fibroblasts were plated in a T75 flask with 50 ml of complete DH medium for 2 to 3 days. The Fibroblast-conditioned DH medium was filtered with 0.22 μm filter prior to use and supplemented with 1 × B-27. Each adult spinal cord glia were plated in 10 ml of Fibroblast-conditioned DH medium on a T75 flask coated with 10 μg ml^−1^ poly-D-lysine. Glia were incubated in a humidified 37 °C incubator with all myelin debris for 4 d without moving it. After 4 d, glia and gamma MNs were washed twice with complete DH medium and newly Fibroblast-conditioned DH medium was added three times per week until confluence. To optimize MNs long-term survival and to obtain high MNs purity, Fibroblast-conditioned DH medium was used instead of complete DH medium in the Motor neuron medium. In order to eliminate any residual glial cells in the MN culture, MNs were treated 24 h after plating with 10 μM cytosine β-D-arabinofuranoside hydrochloride (Sigma-Aldrich) for 48 h or cells were treated with 0.25% trypsin as MNs are more firmly attached on substrate than glia. After reaching confluence, cells were separated from each other. Medium was kept for microglia culture and cells were treated with trypsin 0.05%, EDTA (0.1%) (VWR) at room temperature, which allows astrocytes to detach with few gamma MNs but no microglia. Adult astrocytes were transferred in a tube with complete medium, counted and centrifuged at 300 g for 10 min. The pellet was resuspended in Fibroblast-conditioned DH medium supplemented with 2% B-27 at 500,000 cells per T75 flask coated with 10 μg ml^−1^ poly-D-lysine. Purified microglia medium containing astrocytes-conditioned DH medium was filtered on 0.22 μm filter mixed with a ratio one-to-one with complete DH medium and supplemented with 1 × B-27, 40 ng ml^−1^ basic FGF (Cell Sciences), 20 ng ml^−1^ EGF (Austral Biologicals) and 5 ng ml^−1^ GM-CSF (Invitrogen) to the remaining attached adult microglia and gamma MNs. Adult microglia may take up to 1 month before beginning to proliferate. Medium was changed thrice a week. When microglia have reached confluence, they were purified from remaining cells using 3 steps percoll gradient (Sigma-Aldrich)^31^. Cells were treated with 0.25% trypsin, EDTA (0.1%) and transferred in a tube containing complete DH medium and centrifuged at 300 g for 10 min. The pellet was resuspended in 10 ml of 70% isotonic percoll, overlaid with 20 ml of 50% isotonic percoll and overlaid with 10 ml of HBSS1×. The gradient was centrifuged at 1200 g for 45 min in a swinging bucket centrifuge without brake. Microglia were recovered at the 50–70% interphase, transferred in a new tube and filled with complete medium. Cells were counted and centrifuged at 300 g for 10 min. The pellet was resuspended in Purified microglia medium and plated at 1,000,000 cells per T75 poly-D-lysine coated flask. When astrocytes were removed from the glia flask, gamma MNs can be enriched but survived better when they are mixed with glia cells. Gamma MNs were detached using 0.25% trypsin - EDTA (0.1%) when present with glia, this step was watched under a microscope to stop the reaction when the gamma MNs detached but not the microglia cells. They were kept in the same media as astrocytes.

### Antibodies

Mouse anti-TUJ1 1:1000 (MMS-435P-250, Covance), rabbit anti-CHAT 1:1000 (AB 143, Millipore), mouse anti-NFM 1:500 (MAB1621, Millipore), rabbit anti-ISLET1 1:1000 (20670, Abcam), rabbit anti-GFAP 1:1000 (AB5804, Millipore), rabbit anti-AQP4 1:100 (AB3594, Millipore), rabbit anti-EAAT2 1:100 (ab41621, Abcam), mouse anti-GS 1:100 (MAB302, Millipore), mouse anti-O4 1:1000 (MAB345, Millipore), rabbit anti-IBA1 1:500 (019-19741, Wakochemical), rat anti-CD11b 1:200 (550282, BD pharmingen), mouse anti-MAP2 1:500 (MAB3418, Millipore), mouse anti-TDP-43 1:300 (gift from Dr J-P Julien’s laboratory), mouse anti-NeuN 1:500 (MAB377, Millipore), mouse anti-CNPase 1:200 (NE1020, Millipore), mouse anti-Nestin 1:200 (AB6142, Abcam), mouse anti-SOX2 1:500 (MAB4343, Millipore), mouse anti-Vimentin 1:1000 (AB28028, Abcam), rabbit anti-OLIG2 1:500 (AB9610, Millipore), rat anti-CD31 1:500 (550274, BD Biosciences), rabbit anti-cleaved caspase 3 1:200 AB3623, Millipore), Alexa fluor 488 goat anti-rabbit (A-11008, Life Technologies), Alexa fluor 594 goat anti-mouse (A-11005, Life Technologies)

### Flow cytometry analysis

400,000 E-14.5 MNs collected from individual spinal cord were transferred into 2 × 15 ml collection tubes and centrifuged at 300 g for 10 min at room temperature. Pellets were resuspended in 100 μl of cold PBS. Cells were fixed with 100 μl of 4% PFA (Electron microscopy sciences) for 30 min at 4 °C and washed by adding 1 ml of cold PBS and centrifuged at 300 g for 10 min 4 °C. The supernatant was discarded, this washing step was repeated and the pellet resuspended in 100 μl of immunofluorescence staining buffer containing triton X-100 (0.3%) in 50 mM potassium PBS (KPBS) with 5% goat serum with or without primary ISLET1 antibody (dilution 1:1000) for 1 h at 4 °C. Cells were washed by adding 1 ml of cold immunofluorescence staining buffer and centrifuged at 300 g for 10 min 4 °C. Cells were stained with 100 μl of goat Alexa fluor 488 anti-rabbit antibody (dilution 1:500) in immunofluorescence staining buffer and incubated at room temperature for 1 h. Cells were washed by adding 1 ml of cold immunofluorescence staining buffer and centrifuged at 300 g for 10 min 4 °C. MNs were resuspended in 50 μl of PBS1× for flow cytometry analysis and nucleus were stained with DRAK1. The number of ISLET1 positive cells was corrected with false positive staining observed with unstained control motor neurons using FACS Calibur (BD Biosciences).

### Immunofluorescence

After 2 to 3 days of culture, cells were fixed with 4% cold PFA for 20 min at 4 °C, washed twice with cold PBS 1× and incubated in immunofluorescence staining buffer for 20 min at room temperature. Cells were stained overnight at 4 °C with primary antibodies diluted in immunofluorescence staining buffer and were washed twice with KPBS for 5 min than incubated for 1 h at room temperature with secondary antibodies (diluted 1:500) in immunofluorescence staining buffer and finally were washed thrice with KPBS before mounting with fluoromount G with Dapi (Electron microscopy Sciences).

### MNs purity and density/viability test

MNs were seeded in Motor neuron medium on poly-D-lysine coated 9 × 9 mm cover glass in a 24-wells plate and cell evaluation was done using a 20× objective and a Zeiss axio imager M2 microscope. Extracted E14.5 MNs purity was determined by calculating the ratio of Tuj1/Islet1 or Tuj1/Chat positive cells for each extraction over the number of unstained but Dapi-positive cells after 2 d of culture. For each MN extraction, 4 random fields were selected for purity evaluation. For MNs density/viability test, purified E14.5 MNs were plated at 200,000 cells/cm^2^, 100,000 cells/cm^2^, 50,000 cell/cm^2^, 25,000 cells/cm^2^ and 10,000 cells/cm^2^ for 1, 3 and 6 days. Each density condition was replicated 6 times to increase statistical power. Cells were treated with CAF once after 24 h of culture to avoid glial cells proliferation and 75% of the medium was changed each 48 h. Cells were fixed and stained as mentioned above using Tuj1 or cleaved caspase 3 antibodies. The number of MNs was counted by taking 10 pictures covering all the cover glass for each sample. The percentage of MNs viability was determined by calculating the total number of counted cells/nuclei using DAPI filter per cm^2^ and by dividing it by the number of cells initially seeded per cm^2^ multiplied by 100 for each picture taken. In parallel, the percentage of MNs viability was also determined by counting the total number of cleaved caspase 3 positive cells per cm^2^ and dividing it by the number of cells initially seeded per cm^2^ multiplied by 100 for each picture taken.

### MN transfection

Freshly extracted Hlxb9-GFP positive E14.5 MNs were plated at 800,000 cells on poly-D-lysine coated 9 × 9 mm cover glass in 24-well and cultured in Motor neuron medium. The day after, 200 ng of reporter plasmid was mixed with 40 μl of neurobasal medium and 2.4 μl of plus reagent solution (Life Technologies) was added and mixed together by pipetting the entire volume up and down. The preparation was incubated at room temperature for 15 min. In the meantime, 2.4 μl of lipofectamine was mixed with 40 μl of neurobasal medium in a second micro tube by pipetting the entire volume up and down. After 15 min the micro tube content of one tube was mixed with the other by pipetting up and down the entire volume and incubated at room temperature for 15 min. During this incubation period, the Motor neuron medium in each plated well was discarded and 160 μl of warm Neurobasal A medium was added to each well. The previously prepared plasmid and 85 μl lipofectamine solution was added by droplets close to cells. For this, the plates were tilted at a 45 ° angle to expose one half of the cells on which half of the DNA preparation was added. The plate was gently tilted for the second half of the cells and the same procedure was repeated. Cells were incubated in the incubator at 37 °C for 3 h at 5% CO2. Then, 250 μl of Motor neuron medium was gently added in each well and cells were incubated at 37 °C for 16 h at 5% CO2. Cells were then washed twice with warm Neurobasal A medium and 1 ml of Motor neuron medium was added. Cells were incubated at 37^ °^C for subsequent experiments.

### Permeability test

The capacity of astrocytes to diminish endothelial cell permeability was assessed with *in vitro* vascular permeability assay (Millipore) as manufacturer instruction except for the following modifications. 50,000 astrocytes were seeded on the backside of the transwell. For this, transwells were inverted and cells were allowed to attach for 2 h before plating endothelial cell within each well as manufacturer’s instruction. Cells were maintained for three days in 1:1 Glia and small motor neurons medium with endothelial EBM2 medium before the permeability test was performed according to manufacturer instructions.

### Three-dimensional cellular interaction

MNs were cultured on the top of a fibroblast-populated chitosan/collagen sponge as previously described^33^. For interaction of astrocytes with endothelial cells, two weeks before seeding adult astrocytes, the sponge was overlaid with 600,000 Huvec cells and 1000,000 human fibroblasts in half endothelial EBM2 medium mixed with half complete DH medium supplemented with 50 μg ml^−1^ ascorbate^32^. After 2 weeks, 600,000 adult astrocytes were seeded on top of the sponge and allowed to interact for 28 d in half glial cell medium and half endothelial EBM2 medium supplement with 50 μg ml^−1^ ascorbate. For interaction of adult astrocytes with MNs, two weeks before seeding astrocytes and MNs, the sponge was overlaid with 800,000 human fibroblasts in complete DH medium supplement with 50 μg ml^−1^ ascorbate. After 2 weeks, 1 000,000 astrocytes were seeded with 800,000 MNs for 2 weeks in Motor neuron medium.

### RT-PCR

Microglia were plated at 1 million/9.6 cm^2^, treated or not with 1 μg/ml of LPS for 48 hours in DH medium without serum. RNA was extracted with RNA easy kit (Biobasic). cDNA was produced with superscriptIII reverse transcriptase (Life Technologies) and PCR using Jump Start enzyme (Life Technologies). Microglia specific primers for cDNA expression were IBA1:Forward 5′GATTTGCAGGGAGGAAAAGCT, Reverse 5′AACCCCAAGTTTCTCCAGCAT CD11b: Forward 5′ CAGACAGGAAGTAGCAGCTCCTT; Reverse 5′ TGATACCGAGGTGCTCCTAAAACC CX3CR1: Forward 5′ CAGCATCGACCGGTACCTT Reverse 5′ GCTGCACTGTCCGGTTGTT TNFα: Forward 5′CCAGAACTCCAGGCGGTGCCTATGT-3′ Reverse 5′-TACAACCCATCGGCTGGCACCACTA-3′.

### Statistical analysis

Statistical analyses were performed using 2-way ANOVA with fitted residual according to Akaike Information Criterion. Post-Hoc test were conducted using Student T-test with Bonferroni correction (α = 0.05). The probability level was regarded as significant at p < 0.05.

## Additional Information

**How to cite this article**: Beaudet, M.-J. *et al.* High yield extraction of pure spinal motor neurons, astrocytes and microglia from single embryo and adult mouse spinal cord. *Sci. Rep.* **5**, 16763; doi: 10.1038/srep16763 (2015).

## Supplementary Material

Supplementary Information

## Figures and Tables

**Table 1:** Purified cells from single embryo spinal cords [mean ± STDEVA. (n)].

Tissue source	MNs counts ×10^6^	Glial cell counts ×10^6^	MNs purity (%)	FACS ISLET1^+^ cells (%)
E14.5 Hxlb9-GFP	2.86 ± 0.85 (57)	0.88 ± 0.36 (50)	97.75 ± 2.90 (39)	
E14.5 CD-1	2.24 ± 0.88 (41)	1.01 ± 0.64 (41)	95.08 ± 3.63 (50)	93.82 ± 1.02 (20)
E13.5 CD-1	1.69 ± 0.90 (43)	0.59 ± 0.29 (37)		

**Table 2:** Purified MNs from single adult spinal cords [mean ± STDEVA. (n)].

Tissue source	wtSOD1 1 month	G93A 1 m	C57BL6 5 m	Hxlb9-GFP 5 m	G37R 5 m	wtSOD1 5 m	wtSOD1 11 m	C57BL6 12 m	G37R 12 m
**MNs counts ×10**^4^	6,55 ± 2.27 (6)	5,41 ± 0.83 (6)	1,88 ± 0.46 (6)	3,77 ± 0.70 (5)	2,28 ± 0.43 (7)	5,35 ± 2.11 (19)	6,76 ± 2.14 (6)	2,48 ± 1.22 (5)	3,65 ± 2.22 (6)
