# Supplementary material for: High yield extraction of pure spinal motor neurons, astrocytes and microglia from single embryo and adult mouse spinal cord
Source: Sci Rep. 2015 Nov 18;5:16763. doi: 10.1038/srep16763 (PMC4649473; doi:10.1038/srep16763)
Supplement: Supplementary Information [file srep16763-s1.doc]

**High yield extraction of pure spinal motor neurons, astrocytes and microglia from single embryo and adult mouse spinal cord**

Marie-Josée Beaudet, Qiurui Yang, Sébastien Cadau, Mathieu Blais, Sabrina Bellenfant, François Gros-Louis, François Berthod*

Centre LOEX de l’Université Laval, Centre de recherche du CHU de Québec, and Département de Chirurgie, Faculté de Médecine, Université Laval, Québec (Québec), Canada

*To whom correspondence should be sent:

Dr. François Berthod

Centre LOEX de l’Université Laval,

Centre de recherche du CHU de Québec

Hopital de l’Enfant-Jésus, Aile R

1401, 18e rue

Québec (QC) Canada G1J 1Z4

Phone: +1-418-990-8255, ext. 1705

Fax: +1-418-990-8248

E-mail address: francois.berthod@fmed.ulaval.ca


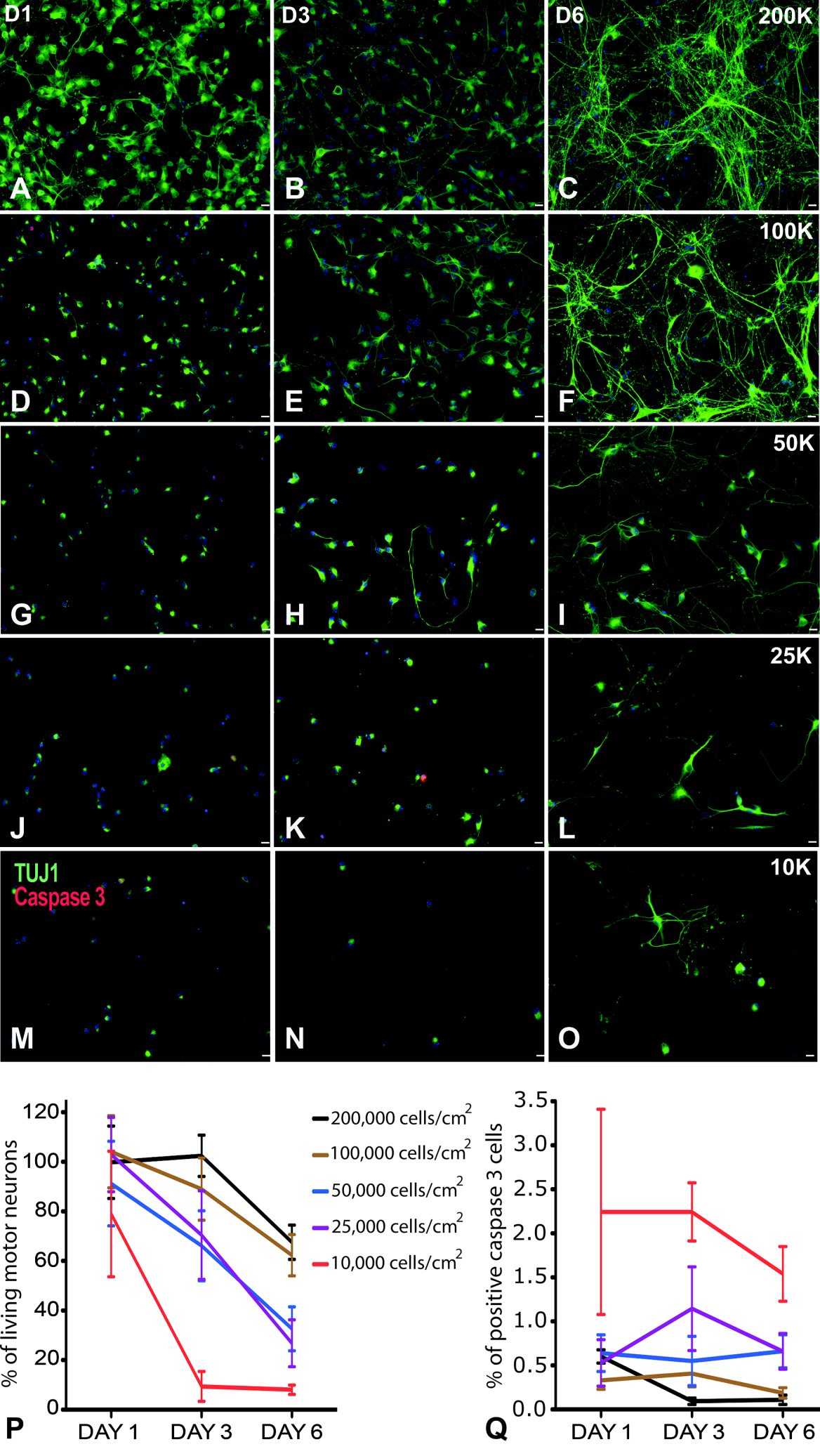


**Supplementary Fig. S1 │**Impact of MN plating density on cell survival. Representative images of E14.5 MNs plated on a 9 x 9 mm poly-D-lysine coated cover glass in Motor neuron medium for 1, 3 and 6 days at densities ranging from 200,000 (A-C), 100,000 (D-F), 50,000 (G-I), 25,000 (J-L) to 10,000 cells/cm^2^ (M-O) are shown. Characterization of MN, extracted from E14.5 CD-1 mouse embryos, by immunofluorescence staining using TUJ1 (in green) and cleaved caspase 3 (in red) cultured for 1 (A, D, G, J, M), 3 (B, E, H, K, N) and 6 days (C, F, I, L, O) can be observed. Bars indicate 20µm.

Quantification of cell survival for each tested plating density over time is also illustrated (P, Q). The total number of counted cell nuclei detected using DAPI expression was divided by the number of cells initially seeded per cm^2^ multiplied by 100 (P). In addition, the total number of apoptotic caspase-3 positive cells divided by the number of cells initially seeded per cm^2^ multiplied by 100 was quantified (Q). No statistical difference between the 200,000 cells/cm^2^ and 100,000 cells/cm^2^ plating densities was detected (*P* > 0.05; n=6). However, a statistical difference was observed, in all other tested conditions, between the 200,000 cells/cm^2^ compared to 50,000, 25,000 and 10,000 cells/cm^2^ plating densities at day 3 (*P* < 0.01; n=6) and day 6 (*P* < 0.001; n=6). Also, statistical differences were observed between the 100,000 cells/cm^2^ versus 50,000 cells/cm^2^ and 25,000 cells/cm^2^ plating densities at day 6 (*P* < 0.001; n=6). For the percentage of caspase 3 positive cells, a statistical difference was observed between the 200,000 cells/cm^2^ plating density and the 10,000 cells/cm^2^ at all days (*P* < 0.001; n=6).

**
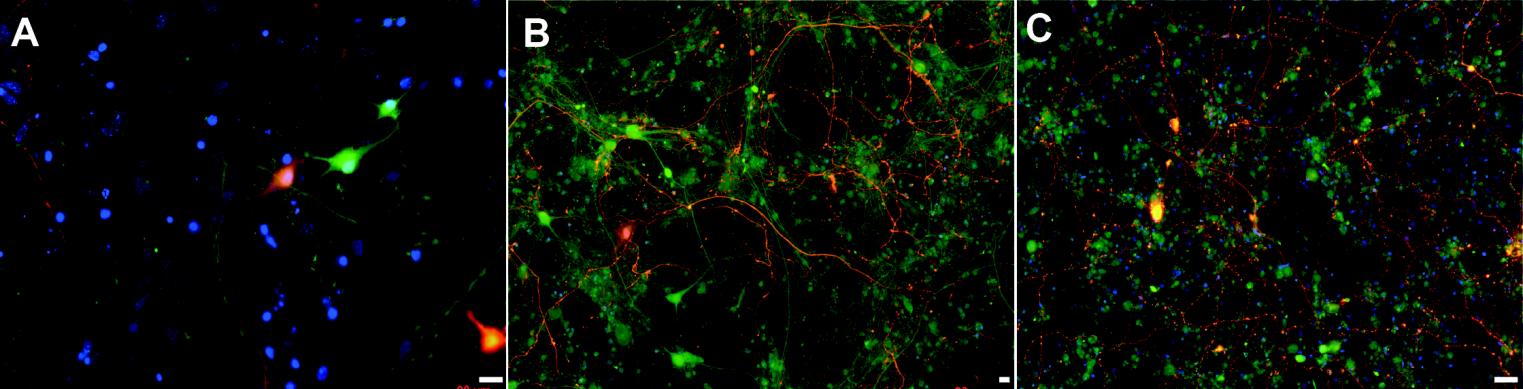
**

**Supplementary Fig. S2 │**Transduction of E14.5 Hxlb9-GFP extracted MNs with pLenti-dsRED plasmid using lipofectamine reagent after 4 (A), 14 (B) and 21 days post transfection (C).


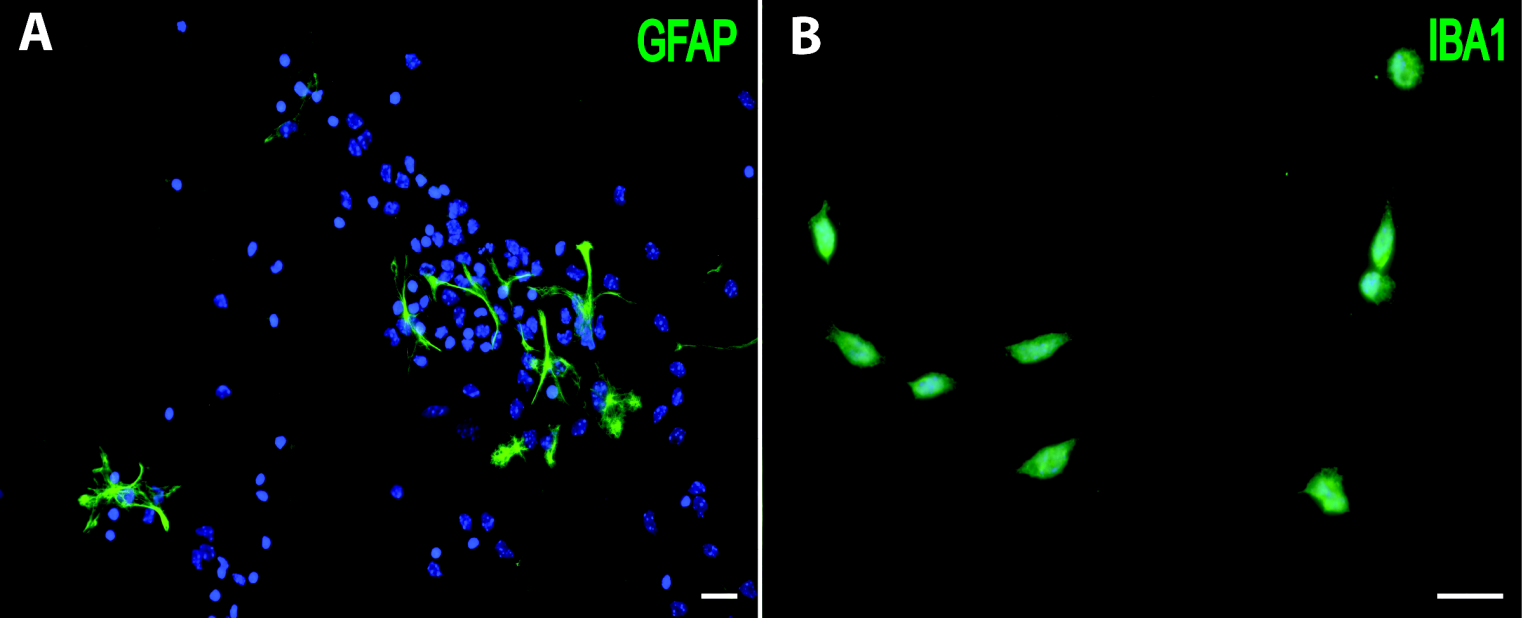


**Supplementary Fig. S3 │** Purified glial cells isolated from one embryonic spinal cord. Characterization using specific GFAP antibody labeling astrocytes (A) or IBA1 antibody labeling microglia (B) of purified glial cells extracted from one 14,5 days old CD-1 mouse embryo and cultured in vitro for 2 days. Bars are 20µm.

***Step by step protocol for motor neurons and glial cells isolation from E14 mouse embryos***

**MATERIALS**

**REAGENTS**

- Leibovitz’s L-15 medium (Life Technologies, cat. no. 21083027) with 25 µg/ml penicillin-gentamycin (Sigma Chemicals cat. no. P3032 and G1264). Note that some laboratories add 0.5 µg/ml fungizone (Sigma Chemicals cat. no. A9528).
- Papain (Worthington, cat. no.LS003119) (see REAGENT SETUP). **CRITICAL** For the motor neuron extraction uses only the 100 mg format of papain otherwise the MNs may die.
- Nycoprep density 1.077 g/ml (Axis-Shield, cat. no, 1077) (see REAGENT SETUP)
- Neurobasal A medium minus phenol red (Life Technologies, cat. no. 12349-015)
- Motor neuron medium (see REAGENT SETUP)
- B-27 supplement (Life Technologies, cat. no. 17504044)
- L-glutamine (Life Technologies, cat. no. 21051-024). Stock at 48 mM in HBSS 1x. Store 5 ml aliquots at -20^◦^ C. Can be kept at 4^◦^ C for one week.
- Hydrocortisone (Calbiochem, cat. no.386698). Stock at 200 µg/ml. Store 1 ml aliquots at -20^◦^ C. Can be kept at 4^◦^ C for one week.
- Insulin (Sigma Chemicals, cat. no. 11882). Stock at 5000 µg/ml. Store 0.5 ml aliquots at -20^◦^ C. Can be kept at 4^◦^ C for one week.
- NaHCO3 (Fisher Scientific, cat. no. S233)
- Human recombinant BDNF (Feldan, cat. no. 1D-14-002b). Stock at 10 µg/ml in water. Store 0.1 ml aliquots at -20^◦^ C. Can be kept at 4^◦^ C for one week.
- Human recombinant GDNF (Feldan, cat. no. 1D-14-014b). Stock at 10 µg/ml in water. Store 0.1 ml aliquots at -20^◦^ C. Can be kept at 4^◦^ C for one week.
- Human recombinant NT-3 (Feldan, cat. no. 1D-14-004b). Stock at 10 µg/ml in water. Store 0.1 ml aliquots at -20^◦^ C. Can be kept at 4^◦^ C for one week.
- Human recombinant CNTF (Feldan, cat. no. 1D-14-016c). Stock at 25 µg/ml in water. Store 0.01 ml aliquots at -20° C. Can be kept at 4° C for one week.
- HBSS 10x without magnesium, calcium, phenol red (Life Technologies, cat. no. 14185-052)
- Embryos from CD-1 Mice or transgenic Hxlb9-GFP mice (Jackson Laboratories)
- Human dermal fibroblasts
- DME-Ham 3:1 medium (DH medium) for cells culture (Life technologies, cat. no.12800 and 21700) (see REAGENT SETUP)
- L-adenine (Sigma Chemicals, cat.no.A2786)
- cytosine β-D-arabinofuranoside hydrochloride (CAF) (Sigma-Aldrich, cat no. C6645-25MG)
- Lipofectamine LTX and Plus Reagent (Life Technologies, cat. no. A12621)
- Fetal calf serum (Hyclone, Logan, UT, cat. no. SH30396)
- Poly-D-lysine (Sigma Aldrich, cat. no. p7280-5MG). Stock at 50 µg/ml with sterile deionized water; see REAGENT SETUP.
- 70% ethanol (to wash surfaces and to wet mouse skin and fur in order to avoid contamination).
- Antibodies for immunostaining: mouse anti-TUJ1 (1:1000; Covance, cat. no. MMS-435P-250), rabbit anti-CHAT (1:1000; Millipore, cat. no.AB 143), mouse anti-NFM (1:500; Millipore, cat. no. MAB1621), rabbit anti-ISLET1 (1:1000; Abcam, cat. no. 20670), rabbit anti-GFAP (1:1000; Millipore, cat. no. AB5804), rabbit anti-IBA1 (1:500; Wakochemical, cat. no. 019-19741), rabbit anti-cleaved caspase 3 (1:200; Millipore, cat. no. AB3623)
- Fluoromount G with Dapi (Electron microscopy Sciences, cat. no. 17984-24)
- Goat serum (Life Technologies, cat. no. 16210-064)
- KPBS buffer solution (see REAGENT SETUP)
- Potassium phosphate monobasic (Fisher Scientific, cat. no. P285500)
- Potassium phosphate dibasic (Fisher Scientific, cat. no. BP363-500)
- Sodium chloride (Sigma Aldrich, cat. no. S5886-1KG)
- Triton x-100 (Sigma Aldrich, cat. no. T8787-100ml)
- Immunofluorescence staining buffer (see REAGENT SETUP)
- Paraformaldehyde (PFA) vials 16% (Electron microscopy sciences cat. no.15710)
- Phosphate buffer saline (see REAGENT SETUP)

**EQUIPMENT**

- 12-well polystyrene plates (BD Falcon, cat. no. 0877229). 1 embryonic spinal cord/well.
- 24-well polystyrene plates (BD Falcon, cat. no. 087721). 400 000 cells/well for immunostaining.
- Cover glass 9x9 mm
- Sterile 20 cc syringe (Terumo)
- Sterile syringe filter unit 0.22 µm (Millipore, cat. no. SLGSM33SS)
- Carbon dioxide chamber for euthanizing mice. **CRITICAL** To achieve a maximum MNs extraction yield and to obtain high quality of extracted MNs, mice should not be anesthetized with isoflurane prior to carbon dioxide.
- 100 mm cell culture dish (Fisher cat. no. 0875712)
- Humidified incubator with 5% CO2.
- Sterile hood for cell culture
- Binocular stereo microscope
- Open sterile hood
- Zeiss axio imager. M2 microscope
- Nikon eclipse E800 confocal microscope and Nikon EZ-C1 software (Nikon, Tokyo, Japan)
- Beckman coulter Allegra 6R swinging bucket centrifuge
- Beckman coulter Z2 particle count and size analyzer
- Hemacytometer
- Transfer pipettes (VWR, cat. no. 414004-015)
- Scissors, micro-scissors
- Sterile paper sheet
- 50 ml polypropylene tubes (BD Falcon, cat. no. 1443222)
- Cell strainer 40 µm (BD Falcon cat. no. 352340)
- Filter unit 0.22 µm
- Serological pipette 10 ml (BD Falcon, cat. no. 1367520)
- Micropipette 1000 µl
- Water bath with controlled temperature at 37^◦^ C
- Sterile 250 ml bottle
- Platform rocker
- Curve forceps
- 1.5 ml tube
- FACS Calibur (BD Biosciences)

**REAGENT SETUP**

**Papain working solution**. Each embryonic spinal cord is digested individually in 1 ml of papain solution (2 mg/ml). The papain solution is prepared just prior to tissue extraction. Dissolve papain in 60% HBSS1x (dilute HBSS 10x with sterile deionized water) mixed with 40% L-15 medium. Note that 10 ml of papain working solution should be prepared to harvest 9 mouse spinal cords, by adding 6 ml of HBSS 1x to 4 ml of L-15 containing 20 mg of papain. To dissolve papain, put the solution in a water bath at 37^◦^ C for 10 to 15 min or until no visible particles are observed into the solution. The solution must also be filtered using a 0.22 µm filter and kept at 4^◦^ C until tissues are ready to be digested. Prior to digestion, warm the papain working solution at 37^◦^ C.

**Nycoprep density gradient.** In order to separate motor neurons from the other cell types, prepare 10 ml of a 1.06 g/ml dilution of Nycoprep just prior to tissue extraction for each embryonic mouse spinal cord. To prepare 10 ml of Nycoprep 1.06g/ml, mix 9.84 ml of Nycoprep 1.077 g/ml with 0.16 ml of L-15 medium in a sterile bottle. Invert the bottle a few times to homogenize the Nycoprep solution. Keep at 4^◦^ C without disturbing. While you gently pour the cells on it without disturbing the gradient, the Nycoprep can stay at room temperature.

**Motor neuron medium**. Motor neuron medium is obtained by mixing complete DH medium with neurobasal A medium and other essential additives. To prepare complete DH medium, use 100 ml of DH medium supplemented with NaHCO3 36.54 mM, L-adenine 0.18 mM, 2N HCL 312.5 µl/L, 10% of fetal calf serum and 25 µg/ml of penicillin-gentamycin. The completed DH medium is then mixed in a one-to-one ratio with neurobasal A medium. Supplement with B-27 1%, L-glutamine 0.24 mM, hydrocortisone 0.2 µg/ml , insulin 2.5 µg/ml , NT3 10 ng/ml, GDNF 10 ng/ml , BDNF 10 ng/ml and CNTF 25 ng/ml. The medium can be kept for one week at 4^◦^ C but the growth factor must be added fresh hidden from light.

**Fibroblast-conditioned DH medium.** To improve motor neuron survival beyond 5 days of culture, MNs should be cultured in a fibroblast-conditioned DH medium. To prepare it, 6 millions of human fibroblasts are plated in a T75 flask with 50 ml of complete DH medium for 2 to 3 days. The fibroblast-conditioned DH medium must be filtered prior to use in order to avoid residual fibroblast contamination. To optimize MNs recovery and to obtain high MNs purity, it is recommended to use the fibroblast-conditioned DH medium only after culturing the cells for 1 week in Motor neuron medium. This will reduce the proliferation of contaminating astrocytes and microglia. In order to eliminate any residual glial cells, it may be useful to treat cells 24 h after plating with 10 μM cytosine β-D-arabinofuranoside hydrochloride for 48 h or to treat cells with trypsine 0.25% as motor neurons are more firmly attached on substrate than glia.

**Poly-D-lysine coating**. In order to favor the attachment of motor neurons and glial cells to the flasks, plastic must be coated with poly-D-lysine (10 µg/ml) prepared in sterile deionized water. Usually, 0.1 ml of poly-D-lysine solution per cm^2^ is sufficient to cover the surface. The poly-D-lysine coating step must be done at room temperature for a 2 h duration period. At the end of 2 h coating period, plates or wells must be washed twice with sterile deionized water. Make sure to cover completely the surface of the well with sterile deionized water and aspirate it completely after washing. The poly-D-lysine coated plates remain stable for 1 week and must be kept dry at 4^◦^ C until use.

**Phosphate buffer saline (PBS)** **1x**. To prepare 1 liter of PBS 1x (NaCl 137 mM, phosphate 10 mM, KCl 2.7 mM), mix in sterile deionized water 8g of NaCl, 0.2g of KCl, 1.44g of Na_2_HPO_4_ and 0.24g of KH_2_PO_4._ The pH of the PBS 1x solution must be adjusted to 7.4.

**KPBS buffer solution**. To prepare 1 liter of 50 mm potassium PBS (KPBS), mix in sterile deionized water 0.45g of potassium phosphate monobasic, 3.8g of potassium phosphate dibasic and 8.1g of NaCl.

**Immunofluorescence staining buffer.** To prepare immunofluorescence staining buffer, add triton X-100 (0.3%) to KPBS buffer solution and 5% serum. Note that the serum must have been produced in the same host as the secondary antibody.

**PROCEDURE**

**Before cell extraction**

MNs and glial cells must be cultured on a poly-D-lysine coating plates. including cover glasses prior to experiments. Make sure that the poly-D-lysine covers the entire surface.

**The day of cell extraction**

**Preparation for embryos isolation. TIMING Less than 10 min**

**1│** The number of pregnant mice can be planned according to an expected yield of 1 to 2 millions of motor neurons per spinal cord obtained from one embryo.

**2│**All the procedure should be done under a sterile laminar flow hood at all time. To save time during the procedure, it is recommended to place under the laminar flow hood four 100 mm culture Petri dish containing 15 ml of cold L-15 medium for each pregnant mice to be euthanized. These Petri dishes will be needed to wash embryos prior MNs extraction. Another set of 100 mm culture Petri dishes will also be needed to keep embryos prior MNs extraction. It is also recommended to place under the laminar flow hood a 24-well plate containing 1ml of cold L-15 medium in each well to recover separately mouse spinal cords in each well. If genotyping of the embryos is needed, you may also use a second corresponding 24-well plate containing 1 ml of cold L-15 to recover embryo’s heads for DNA extraction.

**3│**Binocular stereomicroscope and appropriate sterile surgical instruments must also be placed under the laminar flow hood. Usually, 2 scissors, 2 forceps, 1 micro-scissor and 1 scalpel will be needed for the procedure.

**Embryos isolation TIMING 60 min/pregnant female**

**4│**Procede to euthanasia by placing the mice in a carbon dioxide chamber. Open the CO2 valve for approximately 1 or 2 min or until the death of the animal. To confirm animal death, you may also perform a cervical dislocation after taking out the mice from the CO2 chamber.

**5│**Lay the euthanized mouse under the sterile laminar flow hood on the back of the animal in order to proceed with embryos extraction. Rub the abdomen of the euthanized mouse with ethanol 70% using sterile gauze. Carefully cut and open the mouse abdomen using scissors to reveal the uterus containing embryos. Cut one extremity of the uterus. Using forceps, pull the uterus containing embryos toward the cut extremity starting from the uncut opposite uterus side. Up to 10 embryos per mouse can be isolated.

**6│**Dip the uterus containing embryos in 3 successive 100 mm culture dishes containing 15ml of cold L-15 medium to wash out blood.

**7│**Remove the remaining uterus tissue debris on each isolated embryos with forceps or scissors.

**8│**Transfer the 100mm culture Petri with the embryo on the stage plate of the binocular stereomicroscope and locate the spinal cord.

**9│**Immobilize the embryo with the forceps by grabbing the spinal cord near the hindbrain junction. Using the micro-scissors, remove the embryo’s head and the embryonic tissue surrounding the spinal cord. While exposed, spinal cord must be removed gently from the remaining vertebra by rolling it out instead of pulling it out. This will ensure to recover the intact spinal cord.

**10│** With a scalpel, remove the dorsal root ganglions (DRG) from the spinal cord.

**11│**Using a forceps, transfer the isolated spinal cord in one previously identified well. For genotyping purposes, transfer the remaining embryo’s head in the corresponding well from the second 24-well plate. Isolated embryonic mouse spinal cords are now ready to be transferred in the cell culture room for cell extraction.

**CRITICAL STEP** As transgenic embryos must be genotyped individually, be careful to transfer and label the embryonic mouse spinal cord to the corresponding embryo’s head.

**Cell extraction under sterile hood TIMING More than 30 min**

**12│**Warm the papain solution in a water bath set at 37^◦^C. For each isolated embryonic mouse spinal cord, add 1 ml of papain solution in a new corresponding 24-well plate.

**13│**Using forceps, transfer each spinal cord in the corresponding well containing papain solution.

**14│** Incubate them at room temperature for 10 minutes while dissecting the isolated spinal cord in 4 approximately equal pieces using a scalpel. . Note that a shorter or longer incubation time may have a negative effect on MNs extraction yield.

**15│**Incubate the dissected spinal cord at 37^◦^C for 20 min. Make sure to rotate the plates for 10 sec every 5 minutes. During this 20 minutes incubation period, place a 40 µm cell strainer on top of a 50ml collection tube for each isolated spinal cord. During the last 5 min of incubation, add 5 ml of cold L-15 medium in each cell strainer and let the medium flow through the cell strainer.

**16│**Adjust the speed of the pipetman to moderate flow. Using a 10 ml pipette, add 3 ml of cold L-15 to each digested spinal cord to stop the papain digestion.

**17│**To help the remaining cells dissociate from the tissue, allow the spinal cord and the medium to pass up and down through a 10 ml pipette using a pipetman not more than 6 times. Avoid bubble formation and flow back liquid within the medium. Collect the medium containing only dissociated cells and immediately pour it into the cell strainer.

**CRITICAL STEP** Don’t let cells immobile too long or they will settle down and motor neurons may attach to Petri dishes remarkably fast.

**18│**You can pursue the cell extraction with all the other digested spinal cords. To prevent mixing of cells, it is recommended to use a new pipette for each spinal cord.

**19│**Add 2 ml of cold L-15 to each remaining partially dissociated spinal cord tissue.

**20│**Repeat steps 17 to 19 in order to recover as many dissociated cells as possible.

**21│**Triturate the tissue as step 17 and mix the remaining medium containing dissociated cells with the undigested tissue into the cell strainer.

**22│**Add another 2 ml of cold L-15 to each well to recover residual cells and pour it into the cell strainer.

**23│**Prior to dispose out the pipette from the pipetman, rub any aggregates leftover in the cell strainer until complete dissolution.

**24│**Wash the cell strainer with 3 ml of cold L-15.

**25│**Prior to centrifugation, mix by inversion the 50ml collection tubes 6 times. Centrifuge the 50 ml collection tubes at 4^◦^C and 280g for 10 min using a swinging bucket centrifuge.

**CRITICAL STEP** Should you see a white filament (composed of motor neurons) appearing over the pellet, repeat the centrifugation step in order to completely pellet down motor neurons.

**(A) Motor neurons purification**

**26│**Gently remove the supernatant from the pellet. Be careful to gently aspirate the supernatant near the pellet because it is easy to disturb. You can conserve the supernatant containing glial cells and possibly some motor neurons at this point if needed for additional experiments.

**CRITICAL STEP** Should you need to preserve glial cells, centrifuge the collected supernatant at 4^◦^C and 300g for 10 min. Pellet containing glial cells must be immediately placed at 37^◦^C in complete DH medium, keeping it in cold L-15 markedly decreases glial cells viability.

**27│**Add 1 ml of cold L-15 to the pellet containing MNs and gently homogenize it by up and down aspirations with a p1000 pipette. As MNs tend to aggregate, do not try to over dissociate the aggregates otherwise it may damage cells. Add another 5 ml of cold L-15 medium to the cells.

**28│**Carefully pour the 6 ml containing cells on the surface of the previously prepared Nycoprep solution as indicated earlier in REGENT SETUP.

**CRITICAL STEP** Since motor neurons may easily attach to plastic pipette or pipette tips, it is recommended to use a glass pipette.

**29│**Centrifuge at 900g (2000 r.p.m.) using a swinging bucket centrifuge at 4^◦^C without brake for 20 min.

**30**│Purified MNs will not move into the Nycoprep gradient and stay at the interface between the Nycoprep solution and L-15 medium, while the pellet will contain the glial cells and small motor neurons. Aspirate the interface containing motor neurons with a 10 ml glass pipette. To avoid collecting glial cells and achieve better purification yield, stop collecting the interface prior to reach the pellet (2ml above the pellet is recommended).

**31│**Pour each collected interface containing motor neurons equally in two new 50 ml collection tubes. In order to wash away the Nycoprep solution, complete the volume to 50 ml with cold L-15 otherwise motor neurons won’t pellet down.

**32│**If glial cells are needed, collect the remaining pellet in the centrifuged Nicoprep solution and pool it with the corresponding glial cells from step 26 for each sample.

**33│**You can count the cells at this point, or do it after the next centrifugation step. Use a cell coulter or a hemacytometer. Invert the tube gently at least 10 times before taking an aliquot.

**34│**Centrifuge the motor neurons and the glia cells separately at 425g (1350 r. p. m.) using a swinging bucket centrifuge for 10 min at 4°C.

**Cell plating**

**35│**Plate 400,000 motor neurons per well using a 24-well plate (1.8 cm^2^) containing 2 ml of Motor neuron medium in each well. Glial cells can be plated at various densities in complete DH medium. If motor neurons are to be immunostained, they must be resuspended in 100 µl of medium and plated on the surface of a poly-D-lysine coated cover glass. Let the MNs attach to the coverglass for 30 min at 37^◦^C, then add 2 ml of motor neuron medium to feed them.

**36│**Motor neuron medium should be changes 3 times during the first week of culture. Discard only 75% of the volume of Motor neuron medium and replenish with fresh Motor neuron medium. For longer in vitro culture period, MNs should be kept in fibroblast-conditioned DH medium and change every 2 days. MNs can be kept in culture at least 21 days.

**37│**MNs characterization and cell purity can be carried out by immunofluorescence or flow cytometry analysis (Both ISLET1 or CHAT with TUJ1 should be combined to confirm MN identity).

**Motor neuron transfection**

**38│**Plate 800,000 of freshly extracted MNs in 24-well plate containing cover glass and 2 ml of Motor neuron medium in each well.

**39│**The day after, mixed in a micro tube 200 ng of plasmid with 40 µl of neurobasal medium and add 2.4 µl of plus reagent solution, included with lipofectamine reagent kit, for each plated well. Mixed the micro tube by pipetting the entire volume up and down.

**40│**Incubate at room temperature for 15 min.

**41│**In the meantime, add 2.4 µl of lipofectamine with 40 µl of neurobasal medium in a second micro tube. Mixed the micro tube by pipetting the entire volume up and down.

**42│**After the 15min incubation time, transfer the micro tube content from step 39 into the micro tube containing lipofectamine from step 41. Mixed the pooled micro tube content by pipetting up and down the entire volume.

**43│** Incubate at room temperature for 15 min.

**44│**During this incubation period, discard Motor neuron medium in each plated well from step 38 and add 160 µl of warm Neurobasal A medium the each well.

**45│**Add the previously prepared plasmid and lipofectamine (~85µl) (further named “the DNA preparation”) from step 42 and 43 on top of motor neurons.

**CRITICAL STEP** The DNA preparation must be added on nude MNs, i.e. without medium covering them. Tilt the plate at a 45^◦^ angle to expose one half of the cells and place the extremity of a pipette very close to the cells. One drop at the time, pipette down half of the DNA preparation. Gently tilt the plate for the second half of the cells and repeat the same procedure .

**46│**Incubate cells in the incubator at 37^◦^C for 3h at 5% CO2.

**47│**After the 3 h incubation time, add gently 250 µl of Motor neuron medium in each well containing cells and incubate at 37^◦^C for 16h to 18h at 5% CO2.

**48│**Wash cells twice with warm Neurobasal A medium and add 1 ml of Motor neuron medium. Incubate at 37^◦^C for conducting your experiment and change medium as mentioned in 36.

**TROUBLESHOOTING**

Troubleshooting and solutions can be found in **Table S1**.

**(B) Glial cells purification**

(i) For glial cells with small motor neurons, the pellet (step 26) is resuspended in 10 ml of glia and small motor neurons medium. The cells of the entire adult spinal cord are plated in one T75 flask coated with 10 µg/ml poly-D-lysine. If small motor neurons are needed more than glial cells, pool 2 spinal cords cells in 1 T75 and digest them with 6 ml of the 100 mg format of papain instead of the 1 g format of papain which lead to enhance the ratio of MNs to glia cells.

(ii) Cells are incubated in an humidified, 37^◦^C incubator with 5% CO2.

**CRITICAL STEP** For the first 4 days, the flasks must not be moved neither the medium changed.

(iii) After that, change medium with fresh Glia and small motor neurons medium 3 times per week by adding 15 ml/T75. **Figure 2** shows astrocytes with small MNs and microglia obtained in culture before purification.

**CRITICAL STEP** Don’t let astrocytes reach over confluency. Adult microglia are very difficult to maintain in culture.

(iv) When the flask is confluent, aspirate the supernatant without washing the cells.

(v) To separate microglia from astrocytes, take out medium without washing cells and add 2 ml of trypsine 0.05%, 0.1% EDTA at room temperature, this allows astrocytes to detach but not microglia.

(vi) Neutralize the trypsine with 5 ml of complete DH medium. Aspirate astrocytes and put them in a tube. Take a sample to count them. Add purified microglia medium to the remaining attached microglia. They may take up to 1 month before beginning to proliferate.

(vii) Centrifuge astrocytes at 300g in a swinging bucket centrifuge.

(viii) Resuspend the astrocyte pellets in Glia and small motor neurons medium. Plate 500,000 cells/T75, they are confluent at 2000,000 cells. They can be maintained for long term culture, they can be passed at least 4 times and they can be frozen. There can be small motor neurons and few microglia left. To eliminate them, add 5% of Hyclone fetal calf serum to the Glia and small motor neurons medium.

**CRITICAL STEP** After thawing astrocytes, add 5% of fetal calf serum to the Glia and small motor neurons medium to maintain their viability for the first week.

(ix) If there is still some mixing of astrocytes in microglia culture, when microglia are abundant, purified them with percoll gradient. Wash cells with DH medium and pass cells with 2 ml of trypsine 0.25% added with 0.1% EDTA to detach all of them and neutralize with 5 ml of complete DH medium.

(x) Centrifuge at 300 g, 10 min, in a swinging bucket centrifuge.

(xi) Resuspend the pellet in percoll 70% as mentioned in REAGENT SETUP.

(xii) Over this first phase, add gently 20 ml of percoll 50%.

(xiii) Finally, overlay 10 ml of HBSS 1x on top of gradient.

(xiv) Centrifuge the gradient at 1200g in a swinging bucket for 45 min. The microglia locates between phases 50% and 70%.

(xv) Take out the upper phases and keep the microglia. Dilute this suspension with complete DH medium up to 50 ml and invert tubes a few times.

(xvi) Centrifuge at 300g for 10 min in a swinging bucket centrifuge and resuspend the pellet in Purified microglia medium at 1 000,000 cells/T75 until confluence.

**(C) Small motor neurons purification**

(i) After step (**B**) when mixed cells are 75% confluent, pass them with trypsin 0.05%, 0.1% EDTA. When astrocytes begin to detach, aspirate them and put them in a tube to neutralize them by adding 5 ml of complete DH medium. Wash the flask with 5 ml of DH medium without serum to eliminate residual floating astrocytes and pool it with the first part.

(ii) Pass small motor neurons with 2 ml of trypsine 0.25% added with 0.1% EDTA to detach them and watch them under a microscope to stop the reaction before microglia detach, and neutralise with 5 ml of complete DH medium. Aspirate supernatant and transfert it in a tube. Wash the flask with another 5 ml of medium. It is better to have some glia left with small motor neurons to enhance their survival.

(iii) Take an aliquot to count cells.

(iv) Centrifuge at 300 g, 10 min, in a swinging bucket centrifuge.

(v) Resuspend the pellet to have 100,000 small motor neurons/cm^2^.

(vi) Maintain small motor neurons in Glia and small motor neurons medium and change medium twice a week.

**TABLE S1│**Troubleshooting table

| **Step** | **Problem** | **Solution** |
| --- | --- | --- |
| 17, 27, 28 | The MNs extraction yield is low | As motor neurons adhere to plastic surfaces quite rapidly, don’t allow cells to settle down without any movement or rotation. To avoid loosing cells adhering to the walls of plastic pipettes, try to pipette vertically from the collection tube or simply use glass pipettes. Do not try to completely dissociate motor neuron otherwise cell viability will be affected. and aspirate it only in the first part of the pipette to eject it rapidly over the Nycoprep preparation. Separate cells from aggregate only after the Nycoprep gradient. |
| 25, 28, 30 | The MNs purity is low | Just prior to centrifugation, invert tubes at least 6 times to have a homogenous suspension otherwise large glia cells may pellet down with motor neurons. Make sure that the Nycoprep stay at 4^◦^C. Cells must be added gently on the Nycoprep surface to avoid disrupting the gradient concentration. After centrifugation, make sure not to aspirate the pellet when collecting MNs at the interface between Nycoprep and Motor neuron medium. |
| 9, 10, 17 | The MNs viability is low | Avoid deteriorating spinal cords from step 9 and 10 since MNs extracted from intact spinal cord gives better viability rates. The tissues and cells must stay at 4^◦^C at all time. One other important aspect is to gently pipette and triturate MNs without introducing air bubbles. Transfer or add cells only in medium, not directly on the plates otherwise it may damage the cells. When motor neurons are ready to be plated on tissue culture dishes, use cold medium in order to avoid thermal shock. |
| (B) | Astrocytes have immature square like shape | The mature astrocytes are maintained alive when the medium is properly conditioned by fibroblasts. So make sure that the medium is turning to dark orange. When medium is not enough conditioned, immature astrocytes proliferate and viability of mature astrocytes decreases. |
| (B) | Microglia do not grow | Microglia are very hard to maintain alive for long term culture. To maintain them alive you can condition the Glia and small motor neuron medium on new born pups microglia. When astrocytes are with microglia, do not allow astrocytes to become over confluent unless microglia will detach from flasks and die. |
| 12, (C) | Small motor neurons yield is low | The yield of those cells varies from one spinal cord to another. In order to increase their number, pool 2 or 3 spinal cord per T75 which have been digested individually with 3 ml of papain (from100 mg format powder). |
